# Bipolar Spectrum Disorders in Male Youth: The Interplay between Symptom Severity, Inflammation, Steroid Secretion, and Body Composition

**DOI:** 10.3389/fpsyt.2017.00207

**Published:** 2017-10-18

**Authors:** Andreas Walther, Marlene Penz, Daniela Ijacic, Timothy R. Rice

**Affiliations:** ^1^Department of Biological Psychology, Technische Universität Dresden, Dresden, Germany; ^2^Department of Clinical Psychology and Psychotherapy, University of Zurich, Zurich, Switzerland; ^3^Department of Child and Adolescent Psychiatry and Psychotherapy, Psychiatric University Clinic Zurich, Zurich, Switzerland; ^4^Department of Psychiatry – Child and Adolescent Inpatient Service, Icahn School of Medicine at Mount Sinai, New York, NY, United States

**Keywords:** bipolar spectrum disorders, youth, second-generation antipsychotics, obesity, steroid hormones, pro-inflammatory cytokines, longitudinal monitoring, big data

## Abstract

The morbidity and societal burden of youth bipolar spectrum disorders (BSD) are high. These disorders are multisystemic in that adult populations there are clear interactions with inflammatory processes and steroidal physiological systems. There are much less data concerning these areas of study in youth populations with BSD. This is surprising given the association of youth-onset BSD with puberty and its associated physiological changes. In this mini-review, we overview the theoretical role of inflammatory processes and steroidal physiological systems in youth BSD, describe the greater literature in adult populations, detail the literature in youth populations when available, and overview current proposed molecular mechanistic pathways and interaction effects based on the available data. We also attend to the interplay of this complex system with body composition and weight gain, an especially important consideration in relation to the role of second generation antipsychotics as the first line treatment for youth with BSD in major clinical guidelines. A developmental model of early onset BSD for boys is hypothesized with pubertal hormonal changes increasing risk for first (hypo-)manic/depressive episode. The dramatic androgen rise during puberty might be relevant for first onset of BSD in boys. A shift from general hypercortisolism driven by glucocorticoid resistance to hypocortisolism with further disease progression is assumed, while increased levels of inflammation are functionally associated with endocrine dysregulation. The interacting role of overweight body habitus and obesity in youth with BSD further indicates leptin resistance to be a central moderator of the dynamic neurobiology of BSD in youth. The intent of this mini-review is to advance our knowledge of youth BSD as multisystemic disorders with important contributions from endocrinology and immunology based on a developmental perspective. This knowledge can influence current clinical care and more importantly inform future research.

## Introduction

Bipolar spectrum disorders (BSDs) are chronic, progressive, and multisystemic disorders that are a leading cause of disability worldwide (1). BSDs are characterized by their (hypo-)manic and depressive episodes and include bipolar I, bipolar II, as well as subthreshold bipolar disorders, which represent milder but clinically significant syndromes (2). BSD affects up to 6% of the general population, and when subthreshold BSD symptoms are considered, the societal burden is very high (3). For youth, prevalence rates of up to 5% have been reported. Early onset of BSD is associated with delayed diagnosis, delayed therapy initiation, and an overall more severe clinical outcome (1, 4, 5).

There is a peak age of onset for BSD in adolescence at puberty (6–9) suggesting the wisdom of investigation for any causal link between puberty-related changes in steroid hormone concentrations and BSD development (6–9). In adults, BSDs have been shown to be associated with altered steroid hormone concentrations (androgens and glucocorticoids) (10–13); yet, notably, no studies have investigated the relationship between puberty-related changes in steroid secretion and BSDs. In both youth and asdults, BSDs do show increased levels of inflammatory markers (14–16), though there remains no studies, which explore inflammatory interaction effects with other physiological systems including the hypothalamus–pituitary–adrenal (HPA) axis and the hypothalamus–pituitary–gonadal axis. This is potentially problematic if we aim to increase our biologic knowledge of this multisystemic disorder (17). Thus, in this mini-review, we will outline the importance of the study of steroid hormones in combination with inflammatory cytokines in BSD with a focus on male youth.

Bipolar spectrum disorders-related dysregulations of inflammatory processes and steroid secretion might be regarded as preceeding conditions predisposing individuals to BSD, or as the allostatic load representing the cumulative, multisystemic toll of the disease process. In our consideration, we will also outline that the use of second generation antipsychotics (SGAs) in youth with BSD, which is still considered the first line treatment for BSDs, influences both steroid hormone secretion and inflammation. Weight gain and worsening body composition due to SGA treatment might further increase steroid secretion dysregulation and inflammatory processes in youth with BSDs and thus play an important role in BSD course and progression.

## Search Strategy

Initial computerized literature search was conducted in PubMed. The literature search was conducted using the MeSH terms “bipolar disorder,” “mood disorder,” “manic disorder,” “mania,” and the modifiers “youth,” “treatment,” “antipsychotics,” “inflammation,” “CRP,” “IL-6,” “NF-kB,” “steroids,” “cortisol,” “glucocorticoids,” “sex steroids,” “testosterone,” “androgens,” “estradiol,” “body composition,” “frailty,” “muscle mass,” or “fat mass,” The original PubMed search was conducted for references published between January 1, 1950 and February 15, 2017. We additionally searched references of selected studies, reviews, and meta-analyses for further references. To update the literature search, search was repeated on May 15, 2017 and additional databases (Medline and Embase) were searched with the above listed search terms.

## BSD and Inflammatory Markers

The immune hypothesis of BSDs postulates that increased immune activation precipitates manic and depressive episodes *via* multiple physiologic pathways (18). BSDs are associated with increased levels of proinflammatory cytokines in adult populations (14–16). A recent meta-analysis concluded that in acutely ill patients with bipolar mania interleukin-6 (IL-6), tumor necrosis factor-α (TNF-α), soluble interleukin-2 receptor (sIL-2R), and interleukin-1-receptor-antagonist (IL-1RA) are elevated, while a reduction has been identified after BSD treatment for IL-1RA, which is thought to reflect interleukin-1 (IL-1) activity (19). For chronically ill BSD patients IL-6, interleukin-1β (IL-1β), and sIL-2R (acting as counter-regulatory agent) levels were increased in euthymic phases (19). Another recently published meta-analysis concerning C-reactive protein (CRP) in different mood states (mania, depression, euthymia vs. healthy controls) in BSD patients found moderately increased CRP levels for BSD patients in euthymic and depressed states, substantially increased CRP levels during mania, and a reduction of CRP levels in BSD patients achieving euthymia after treatment (20). Interestingly, patients who were not taking medication (SGA and others) had significantly higher CRP levels in manic and depressive states relative to those who were. Supporting evidence for the inflammatory hypothesis also stems from a large Mendelian randomization analysis using a genetic risk score including four single nucleotide polymorphisms in the CRP gene (21). The study showed a 1.21-fold increased risk for bipolar disorder with a 10% increase in genetically determined CRP levels. Anti-inflammatory agents as putative pharmacological intervention against BSD are currently under study, though clear evidence for effectiveness is still lacking (22).

Only one study was identified that explored inflammatory markers in adolescents (23). In this study, IL-6 and high sensitivity C-reactive protein (hsCRP) were explored. Only hsCRP was significantly associated with manic symptom severity (23). The lack of studies in children and adolescents is possibly due to the difficult access to this very high burdened and vulnerable population. To better understand this disorder and to improve treatments, more research is needed to clarify the causal relationship of increased inflammation and BSD in youth and only large prospective studies might answer this question.

A potential molecular pathway explaining these findings and through which inflammation may relate to BSD is the characteristic of CRP in high peripheral concentrations to increase permeability of the blood–brain barrier and thus by entering the central nervous system (CNS) directly to interact with specific CNS cells. CRP is thought to cause hyper-reactivity of microglia and astrocytes. This reactivity leads to intensified immune activation by releasing IL-6 and interferone-γ, activating the tryptotphan degrading enzyme indoleamine 2,3-dioxygenase, increasing quinolic acid synthesis and 3-hydroxy kynurenine. These generate oxidative radicals and decrease brain-derived neurotrophic factor, contributing to neuronal damage (20, 24).

Another possible pathway is the NF-kB signaling pathway, which is commonly activated in inflammatory or autoimmune diseases. This pathway consists of different inducible transcription factors that regulate immune and inflammatory responses. Chronic cellular stress might dysregulate the NF-kB cascade by prolonged activation and subsequent exhaustion and hindering it from its primary function—cell protection from undergoing apoptosis in response to cellular stress (25, 26). The recurrence of mood episodes may induce excessive production of proinflammatory cytokines that exceeds the downregulatory capacity of the inflammatory system and leads to a constant overactivation of microglial cells. Overactivation would promote the secretion of proinflammatory cytokines such as TNF-α and IL-1β, which might directly drive neuronal cell death (27, 28). Supporting findings have been recently reported, showing that regulatory factors of the immunoreaction (IL-23, IL-17, TGF-1β, and TNF-α), have been decreased after 8 weeks of combination pharmacotherapy with lithium and quetiapine in adult bipolar patients achieving response (29). These proinflammatory cytokines have been shown to mediate cell immunity, humoral immunity, and autoimmunity. However, NF-kB has recently received increased attention because of its colocalization in neurons and glial cells and its activation during the induction of long-term potentiation—a paradigm of synaptic plasticity (30). Therefore, NF-kB signaling seems also to be implicated in controlling of axon initiation, elongation, guidance, and dendritic spine density contrasting the abovementioned properties and indicating its important role in learning, memory, and mental diseases due to the modification of neural processes (31).

The presented literature indicates inflammation to be bidirectionally involved in BSD development and progression. Inflammation has further been closely linked to stress and its neuroendocrinology. To what extent steroids are involved in the relationship between inflammation and BSD and whether steroid hormones independently might contribute to BSD development and progression will be outlined in the following.

## BSD and Steroid Secretion

Changes in steroid hormone concentrations have been linked to depression outside the BSD population (32–35), and BSDs are directly associated with altered steroid hormone concentrations (e.g., androgens or glucocorticoids) as well (10–13). For example, postmortem brain tissue analysis reveals differing levels of neuroactive steroids between patients with BSD and healthy controls, suggesting that steroid hormones may be candidate modulators of the pathophysiology of the BSDs and potentially therapeutically relevant (36). More precisely, pregnenolone, which is a progestagen directly metabolized from cholesterol, and dehydroepiandrosterone a subsequently metabolized androgen, were significantly higher in the posterior cingulate and the parietal cortex of patients with bipolar disorder compared to controls. These neuroactive steroids demonstrate physiological relevant concentrations and act on the inhibitory GABA_A_ and excitatory NMDA receptors. Whether steroids are candidate modulators of the pathophysiology of BSDs and which steroids are increased or decreased in youth suffering from BSDs is a matter of current debate.

Hypothalamus–pituitary–adrenal axis dysregulation and altered glucocorticoid function in BSD were reported by Evans and Nemeroff 30 years ago (13, 37). Since this initial investigation dysfunction at multiple levels of the axis has been discovered. Serum cortisol day profiles are elevated in BSD patients over all disease phases including mania/hypomania, depression, and remission as compared to healthy controls (38). Higher chronic cortisol secretion is significantly higher in BSD patients and correlated with manic symptoms (11). Higher free urine and cerebrospinal fluid cortisol levels are found during mania, and many patients with BSDs have attenuated responses to the dexamethasone suppression test (DST) (39). Attenuated responses indicate a dysregulation of the HPA negative feedback and glucocorticoid resistance (40). An increased response to the dexamethasone–corticotropin-releasing hormone test (DEX-CRH) has been reported for acute manic patients compared to healthy controls in another rather small sample (41), as well as for DST only comparing 24 euthymic BSD patients with 26 healthy controls. Studies exploring a more differentiated pattern of HPA axis dysregulation in BSD have found female sex and age of onset to be moderators of DST response, demonstrating the importance of sex and developmental differences (42). Age was negatively associated with DST response in BSD although cortisol is known to continuously increase with age in healthy subjects (33, 43), and long-term use of lithium reduced this association, suggesting that older age is associated with lower HPA activity in BSD. The authors of this study proposed a developmental model of HPA activity in BSD suggesting high stress load in BSD over time would lead to a progression of the HPA axis regulatory status from hypercortisolism and eucortisolism to hypocortisolism. Passos and colleagues point out the anti-inflammatory character of cortisol based on the glucocorticoid receptor (GR) (27). The GR inhibits the NF-kB cascade, which further decreases synthesis of proinflammatory cytokines. Therefore, in BSD-induced glucocorticoid resistance (decreased GR function), both elevated cortisol and inflammation levels are present due to the loss of the anti-inflammaotry properties of the GR (27).

Androgens are present in men up to 10 times higher as in women. The aforementioned differences in BSD with regard to sex might originate from influences of sex steroids on BSD development and progression. Changes in sex steroids have also been linked to depression (32–34, 44, 45). Different studies show altered androgen levels (e.g., testosterone or dehydroepiandrosterone) in BSD. Testosterone positively correlates with the number of manic episodes and the number of suicides when controlled for sex (10), while dehydroepiandrosterone levels are elevated in bipolar patients (36). The hypothesis has been postulated that low testosterone might be associated with psychopathological manifestations such as suicidal behavior in older men, while high testosterone levels might be related to suicidal behavior in adolescents (46). Peak age of onset for BSD is adolescence, suggesting a putative causal link between puberty-related development changes and vulnerability for BSD development (47). Sex-specific endocrine changes are a key determinant for this transition phase, which occurs in boys between 8 and 17 years of age (48–50). Boys experience a high velocity increase of both testosterone and estradiol while girls experience a high velocity increase in estradiol and the testosterone precursor androstenedione (51). However, other lines of research focused on sexually dimorphic fear and aggressive behavior in mice regulated by the biosynthesis of neurosteroids (52, 53). Mice receiving long-term treatment with anabolic androgenic steroids [e.g., testosterone propionate (TP)] showed increased anxiety and aggressive behavior (54). In men, long-term treatment with TP was associated with impulsive aggression, episodic mania, and major depression, while withdrawal was associated with depressive symptoms and suicide attempts indicating sexually different effects of sex steroids (54).

As potential pathophysiology underlying mood disorders, sex steroids have been shown to affect synthesis, metabolism, receptor concentration, and trafficking of various neurotransmitter systems strongly related to mood disorders (44). With regard to estradiol, mixed effects are reported for the serotonin and dopamine system. Estradiol has been shown to reduce serotonin transporter (SERT) mRNA transcription as well as alter the peripheral SERT protein levels and binding and increase serotonin (5-HT_2A_) receptor binding and mRNA (44). Importantly, 5-HT_2A_ activation has been associated with depressive-like phenotypes in a multitude of studies, while 5-HT_2A_ antagonists such as MDL100907 reduce depressive-like behavior (55). Some inconclusive evidence exists that show associations between estradiol, dopamine release, and receptor density in specific brain regions (44). However, these reviews (44, 56, 57) neglect androgen or testosterone action and, not surprisingly, conclude that changes in estradiol levels are associated with mood disturbances. In rodent models, estradiol seems to mediate the antidepressant-like effects of testosterone *via* aromatization of testosterone to estradiol in the dentate gyrus (35). Importantly, serotonergic neurons are only indirectly sensitive to testosterone as they express no androgen receptors (58) and are thus influenced through testosterone aromatization to estradiol and activation of the estrogen beta receptor.

In humans, a negative association between peripheral testosterone—but not estradiol—and global 5-HT_4_ receptor binding has recently been shown for the male brain in 41 healthy men using positron emission tomography (59). Perfalk and colleagues remark that men and women differ for several aspects of the serotonin system as women demonstrate lower 5-HT_4_ receptor binding throughout different parts of the limbic system. Global 5-HT_4_ receptor binding is inversely related to synaptic serotonin levels, indicating increased extracellular transmitter levels to decrease receptor expression and *vice versa* (59). *Post hoc* regional analysis of the data demonstrated that there was a negative association between peripheral testosterone and 5-HT_4_ receptor binding within the hippocampus, while estradiol was positively associated only in *post hoc* analysis to 5-HT_4_ receptor binding within the amygdala (59). As anatomical differences exist in individuals with BSD in the amygdala and, in adolescents, hippocampal volume, these results seem relevant when constructing an informed developmental model (13). Finally, testosterone and estradiol are discussed to influence adult hippocampal neurogenesis and related antidepressant effects attributed to these specific brain areas (60).

Taken together, the reviewed literature shows a considerable amount of studies indicating a bidirectional link between steroid secretion and BSDs with elevated levels of glucocorticoids and androgens in BSDs at an early stage, while there might be a potential switch to hypo-cortisolism and hypo-gonadism with disease progression into older age. However, the underlying pathophysiology is not yet fully understood. Increasing interest has recently been paid to developmental models and the organizational effects of steroid hormone secretion and inflammatory processes on the neurodevelopment with regard to BSDs, what will be discussed in the following.

## Influence of Sex, Steroids, and Inflammatory Processes on Neurodevelopment

While both sexes undergoe a similar pattern of brain development from caudal to rostral and subcortical to cortical-limbic maturation, male fetuses show a slower brain maturation compared to females going along with a slower behavioral development what is expressed immediately after birth (61, 62). Therefore, Schore (63) concludes that the male and female brain display differential sensitivity to early life stress. This may account for why boys are more susceptible to a number of psychiatric disorders, including autism spectrum disorders and attention-deficit hyperactivity disorder. This is supported by research showing for example male newborns being less responsive to auditory and social stimuli and that male newborns are less able to maintain eye contact and show greater difficulties to maintain affective regulation (64). Sex steroids have been shown to play a crucial role in brain development and organization (65). The slower male brain maturation and the greater disinhibition observed in boys has been attributed to prenatal testosterone exposure (66). However, steroids have multiple functions in different brain regions, and androgens and estrogens often display opposing effects (60). For androgens, effects such as facilitation of fetal synaptogenesis and inhibition of synapse elimination leading to slower synaptic maturation as well as influencing of adult hippocampal neurogenesis have been reported (60, 63).

For higher levels of glucocorticoids, detrimental effects on the fetal brain development have consistently been reported (67), while for microglia, the macrophages of the brain, a major role in synaptic pruning during postnatal development has been reported linking microglia surveillance to synaptic maturation. However, neuroinflammation represented by microglial activation is strongly determined by psychosocial stress and chronic elevated stress with subsequent elevated levels of cortisol inducing excessive microglial activation, what might predispose the adolescent brain to BSDs (68).

It is, therefore, well recognized that the developing brain is very sensitive to the effects of circulating steroid hormones and inflammatory markers during sensitive periods of pre-, peri-, and postnatal development, and hormonal surges during critical developmental time windows program behavior and physiology (e.g., testosterone surge in male fetuses during the 8th to 24th week of gestation and again in the first months after birth) (63, 69–72). In addition to the effects of steroids and inflammatory processes on the neurodevelopmental predispostion to BSDs, the pharmacological treatment of BSDs in youth is another relevant contributor to inflammatory and steroidal alterations *via* the subsequent weight gain as discussed in the following.

## BSD Treatment in Youth: SGAs are Considered First Line Treatment

Since the year 2000, prescription and use of SGAs such as quetiapine or olanzapine have risen in adults and in youth (73). SGA use is now the first-line treatment for youth with BSDs and has given clinical benefits in psychiatric stabilization (74). However, this practice is associated with substantial weight gain: at present, approximately 70% of youth treated with SGAs are overweight.

Mechanisms of SGA-related weight gain are considered multifactorial and include endocrine and metabolic factors. Monitoring steroid hormones and specific measures of body composition might be of additional interest to further explore the causality of SGA-induced weight gain (75, 76). SGAs are generally thought to be anti-inflammatory (77), and interesting cross-sectional interactions of SGA use, BMI, hsCRP, and glucose levels have been reported (78). Another longitudinal study investigating a 6-week SGA (clozapine) treatment in psychotic children identified a significant SGA-related increase of a food intake stimulating hormone leptin, raising the question of SGA-induced leptin resistance (76). In a prospective community, cohort study of 339 adults followed over 6 months, a weight gain was observed in 50% of the adults, while food cravings and chronic stress decreased (79). Higher serum cortisol, insulin, and chronic stress were independently predictive of greater future weight gain again indicating leptin resistance as relevant mechanism in BSD progression.

Hypothalamus–pituitary–adrenal axis activation with subsequent glucocorticoid release is thought to increase the intake of highly palatable foods (80). A recent study including 2,557 adults found higher hair cortisol concentrations to be associated with higher BMI, waist-circumference, and obesity (81). Chronic stress, as represented in elevated hair cortisol concentrations, is supposed to cause a disruption of the lipid synthesis leading to accumulation of triglycerides in the liver or adipocyte enlargement. This suggests increased adipocyte proliferation, increases hypothalamic expression of NPY, and finally leading to leptin resistance (82, 83).

Although different strategies to reduce SGA-related weight gain such as healthy lifestyle interventions, switching to lower risk antipsychotics, or combination treatment with weight reducing medication exist, they show limited effectiveness represented by an average weight reduction of around only 3 kg (84). However, metformin—known for its prevention of weight gain and its extreme safety in youth—emerges as potent adjunct for combination treatment with SGAs in youth with BSD (85, 86). Metformin has already been associated with a statistically and clinically significant weight reduction induced by the reduction of hepatic gluconeogenesis and the improvement of glucose uptake as well as its weight stabilizing capacity (87).

Metformin has further been shown to reduce measures of adiposity among overweight and obese adolescents with type 1 diabetes (88), to inhibit androgen biosynthesis (89, 90), and to reduce inflammatory markers such as hsCRP and IL-6 (91–93). The anti-inflammatory medication Celecoxib may also be a promising adjunct as it has been found to promote an accelerated treatment response among acutely ill patients at early illness phase (18). Furthermore, celecoxib has been shown to enhance GR function countering glucocorticoid resistance due to GR dysfunction in BSD as described above (94). Since weight gain has shown to influence BSD progression, a review on the association between body composition and mood disorders will subsequently be presented.

## Body Composition and Mood Disorders

Youth with BSD typically show high rates of overweight (BMI over 25) and obesity (BMI over 30) (95, 96). Overweight and obese youth with BSD display a worse illness course, a higher risk of adult overweight/obesity, and higher overall morbidity (18, 97).

The treatment of youth with BSD, as above noted to be overwhelmingly and increasingly with SGAs, is one contribution to this state. However, changes in puberty are also contributory. In puberty, significant changes in body composition occur and need to be taken into account for the longitudinal examination of youth with BSD (48). A positive association between fat mass and depressive symptoms exists (98, 99). Among many pathways, depressive symptoms may cause lipid dysregulation resulting in increased fat accumulation (82). Extracellular water (ECW) has positive associations with clinical conditions (100), and a recent study linked water intake and mood states in healthy young women (101). There is only one further study demonstrating an association between ECW or water balance and depressive symptoms in men (102), while the examination of the relationship of (hypo-)manic symptoms and body water has never been carried out.

Obesity has been postulated to be an inflammatory state. High levels of inflammatory cytokines have been detected in fat cells, and inflammatory cytokines are also involved in the fat metabolism and are positively associated with obesity (103). Besides HPA axis dysfunction and leptin resistance, adipocyte dysfunction is also suggested as an underlying mechanism of obesity-associated inflammation (104). Declining adipose tissue levels reduce adipose tissue production of TNF-α, IL-6, IL-8, and leptin (105). Importantly, adipose tissue also serves as the main site of the conversion of testosterone to estradiol. In men, around 80% of estradiol is produced in the adipose tissue *via* aromatization (106). Higher testosterone levels are associated with better male body composition (more lean mass, less fat mass) (107), which are also enhanced by testosterone treatment in men (108). Furthermore, as described above, BSDs have been related to a dysregulation of the NF-kB signaling pathway due to prolonged activation. Increased NF-kB signaling leads to increased RANK ligand-induced osteoclastogenesis (109), what might culminate in premature osteoporosis in youth suffering from BSDs. In line with this, Bolton and colleagues showed that mental disorders and related medication use (e.g., antipsychotics, lithium, mood stabilizers) were associated with an increased risk for osteoporosis and fractures in adults (110). This functional cross talk between male body composition, frailty, inflammatory markers, and steroid hormones should be taken into account, when deciding on treatment strategies in male youth with BSD.

## Future Directions and Conclusion

Research on BSD development, progression, and treatment has strongly focused on inflammatory markers. Steroid hormones and the regulatory mechanisms of body composition itself have not yet been sufficiently addressed. The relationship between BSD symptomatology, steroid hormones, and inflammatory markers has mostly been examined in adult samples. Studies investigating the role of changes in steroid hormones, inflammatory markers, and body composition in youth with BSD have never been carried out.

Large prospective studies need to address this open question and should explore interaction effects for this multisystemic disorder (17). A recent review suggests that there may be merit in attempting to combine different biomarkers for better characterization, prevention, and treatment of BSD (111). We agree and in this mini-review have expanded the number of suggested biological systems critical to investigate. However, as a major limitation of this review on BSDs in youth, it is to mention that there are relatively few existing studies, which examined youth with BSDs and inflammatory or steroidal markers. Therefore, this review was complemented with studies on adult BSDs samples reporting levels of inflammatory markers and steroid hormones. Conclusions drawn from these studies might, therefore, not be entirely applicable to youth populations suffering from BSDs. More research on youth suffering from BSD is needed.

Whether dysregulations in the secretion of steroid hormones or inflammatory markers represent predisposing factors increasing vulnerability to BSDs in youth, or if these dysregulations are to be considered measures of allostatic load emerging due to the adaptation to BSDs can only be answered with prospective cohort studies monitoring large samples of children. The insights provided by these suggested study designs will increase the recognition of the relevance of multiple body systems for BSD development, progression, and treatment. Monitoring alterations in steroid secretion and levels of pro-inflammatory cytokines in youth at risk for BSD development presents itself as a promising avenue to better understand the dynamic nature of BSD. Moreover, the information obtained in these studies will help to counterbalance hormonal and inflammatory dysregulations in youth related to BSD and promote tailored BSD treatment in youth.

## Author Contributions

AW conducted the initial review and designed the concept of the Mini Review and wrote the first draft. PM, DI, and TR edited for intellectual content.

## Conflict of Interest Statement

The authors declare that the research was conducted in the absence of any commercial or financial relationships that could be construed as a potential conflict of interest.
